# Effects of Network Characteristics on Reaching the Payoff-Dominant Equilibrium in Coordination Games: A Simulation study

**DOI:** 10.1007/s13235-015-0144-4

**Published:** 2015-02-24

**Authors:** Vincent Buskens, Chris Snijders

**Affiliations:** 1 Department of Sociology/ICS, Utrecht University, Padualaan 14, 3584 CH Utrecht, The Netherlands; 2 Human Technology Interaction Group, Eindhoven University of Technology, PO Box 513, 5600 MB Eindhoven, The Netherlands

**Keywords:** Coordination, Social networks, Dynamic games, Simulation methods

## Abstract

We study how payoffs and network structure affect reaching the payoff-dominant equilibrium in a $2\times 2$ coordination game that actors play with their neighbors in a network. Using an extensive simulation analysis of over 100,000 networks with 2–25 actors, we show that the importance of network characteristics is restricted to a limited part of the payoff space. In this part, we conclude that the payoff-dominant equilibrium is chosen more often if network density is larger, the network is more centralized, and segmentation of the network is smaller. Moreover, it is more likely that heterogeneity in behavior persists if the network is more segmented and less centralized. Persistence of heterogeneous behavior is not related to network density.

## Introduction

While social networks are widely considered as facilitators for cooperation and are judged as instrumental for reaching more efficient outcomes in society [11, 24], not all networks are equally beneficial under all circumstances. When game play occurs on networks, it is not at all obvious to which state—if any—equilibrium behavior will converge. This theoretical and empirical controversy has been most apparent in research based on Harsanyi and Selten’s [18] equilibrium selection principles *payoff dominance* and *risk dominance*. The payoff-dominant equilibrium is the equilibrium that Pareto dominates other equilibria, while the risk-dominant equilibrium is the equilibrium that actors are more likely to choose if they are more uncertain about what the other actor will do. The choice between different equilibria is then considered a matter of coordination on an appropriate convention, more than it is a matter of optimal choice. Several scholars have analyzed theoretically how equilibrium behavior, and thereby the emergence of conventions, depends on differences in the size and shape of the networks between actors (the ‘local interaction structure’), in ways similar to the seminal work of Nowak and May [23] regarding the feasibility of cooperative behavior in the Prisoner’s Dilemma when played on a lattice.

### Theoretical Research on Network Effects in Coordination Games

With respect to coordination games, Ellison [14] has argued that, in general, local (as opposed to random) interaction strongly increases the speed of convergence to the risk-dominant equilibrium. Ellison takes this as evidence in favor of the applicability of evolutionary models to larger populations of locally interacting actors. Anderlini and Ianni [1] likewise show, among other things, how stable states that cannot be reached in a situation where interaction is not local, *can* be reached when local interaction is assumed. Networks in these studies most often have regular structures such as a line [14] or a lattice [4, 5]. What these studies also have in common is that the networks are relatively small in size, and the number of different networks being considered is usually relatively small. The networks that are being compared serve as stylized examples of real- life networks and are mainly meant (1) to show that (different kinds of) network effects on (the speed of) cooperation can exist and (2) to compare theoretical predictions on given networks with empirical behavior in these networks. Although the general gist of this research seemed to be that networks favor cooperation in different contexts, several puzzling findings remained, and more extensive (simulation) analyses have followed, with different research approaches. One prominent research line focuses on which kinds of network characteristics are known to exist in real-world networks and then compares collections of networks with such characteristics with a baseline collection of networks. Analyses along these lines have been conducted in for instance Santos et al. [28], Tomassini and Pestelacci [30] and Roca et al. [25–27]. Santos et al. [28] show that heterogeneity in terms of the degree distribution positively affects cooperation in several games, by comparing populations of single-scale networks with scale-free networks. This positive effect of the degree distribution, however, seems not in line with several other results. Tomassini and Pestelacci [30] compare large random networks, scale-free networks and networks based on a real-world structure. They conclude that only small differences between the different types of networks exist in Stag Hunt games, although community structure does play a role for some network types. Roca et al. [25–27] conduct similar kinds of analyses based on a larger set of underlying games, different kinds of strategy updating rules and a larger set of networks (different variants of random, scale-free and small-world networks). Among many other things not directly related to our paper, they find that ‘best response [network simulation] is largely unaffected by the underlying network, which implies that, in most cases, no promotion of cooperation is found with this dynamics.’ What is well established is that the findings on network characteristics (can) depend on several dimensions, including the strategy update rule, the kind of network and game, the possibility of dynamically rewiring ties, and the game and the choice of payoffs. Typical for research in this direction is that although large classes of (often also large) networks are compared, the focus in terms of network characteristics is on a comparison of one or two network characteristics across groups, with no or less attention for potential effects of other network characteristics. That is, a found difference or a lack of a found difference between, say, a group of random networks and a group of scale-free networks might be due to other characteristics of the networks in the respective groups.

Cassar [10, p. 228], when considering hypotheses about the effects of network characteristics on the kind and speed of convergence, argues ‘it is important to note that these results [on the effects of network characteristics] are exploratory, because a theory linking network characteristics to individual behavior is yet not available. It is hoped that these empirical findings stimulate such theoretical development.’ We argue that, because results in the studies summarized above focus on comparisons of large groups of networks that do not control for possible other structural differences in the networks, the reasons for effects of network characteristics on cooperation (in games in general and coordination games in particular) are incomplete. We try to fill this gap, at least for smaller networks in coordination games. The focus on smaller networks allows us to consider many different networks and to control for the differences in structural characteristics between these networks to a large extent. In addition, the focus on smaller networks allows our results to be directly applicable to empirical research on smaller networks, for instance, on public good provision in small groups or social influence in school classes, or on cooperative behavior in networks in the experimental social sciences.

### Coordinating on Efficient Play as Dependent on Network Characteristics

By simulating game play in a large number of different networks, we analyze how characteristics of networks affect the likelihood of ending up in a specific equilibrium. This is a research technique applied also in some sociological studies (see [7, Chapter 4], [9, 36]). Common to these analyses is the idea that the way in which actors are connected is of key importance for their behavior and that simulation can be used to eventually ‘partial out the net effects’ of the different network characteristics. Moreover, varying the payoff configurations allows us to consider interactions between the payoffs and network characteristics as well. By including a completely deterministic and a more probabilistic version of the simulation, we likewise investigate whether our results change as a consequence of these different setups. Our main research question is how network characteristics such as network size, density, centralization and segmentation affect the probability that locally interacting actors in a network converge to the payoff-dominant equilibrium in a coordination game and how these effects depend on the payoffs of the underlying coordination game. In addition, we examine how those network characteristics determine the probability that the behavior in the network becomes homogeneous (everybody chooses the same behavior) or remains heterogeneous (see also [27]). Compared to earlier theoretical analyses, we cover a larger variation of network characteristics simultaneously, including the density of the network, the variation in numbers of ties between actors (centralization), the clustering of social ties (segmentation) as well as some network aspects (such as the specific number of neighbors actors have) that turn out to have theoretical relevance, but were not identified in earlier analyses. As we will show, the different types of network effects that we encounter are only relevant in a limited part of the payoff parameter space of the games we analyze.

### Empirical Research on Coordination Games

A series of empirical analyses, mainly in experimental economics, has considered the conditions under which human subjects choose which kind of equilibrium in games where both kinds of equilibria exist (cf. early studies by Cooper et al. [12] and Van Huyck et al. [33, 34]), again, often related to theoretical arguments aimed at developing a theory of the evolution of conventions [20, 37]. Unfortunately, the empirical studies do not provide consistent evidence on the empirical importance of network structure to convergence to one or the other equilibrium. Keser et al. [21] found that play in a ring network (each actor has two neighbors) is less likely to lead to the efficient outcome than play in a mixed network. Berninghaus et al. [4] also found that a ring network (of size 8 or 16) leads to less convergence to the Pareto-efficient strategy when compared to groups of three connected players and that lattices of size 16 also lead to efficient play less often. Frey et al. [15] find no evidence of network effects in coordination games (using networks of size 6). In part, this could be due to the fact that the power to find such differences was relatively low given that the more experienced participants quickly converged to the Pareto-optimal solution. Antonioni et al. [2] find that subjects are equally likely to converge to the efficient outcome when comparing a random with a ‘cliquish’ network. The experiments by Cassar [10] showed that coordination on the efficient outcome was more likely in a scale-free network (compared to a random network, both of size 18), possibly because of the higher level of clustering in the scale-free networks. Given that the theoretical results depend on several parameters of the model, as mentioned above, that the variance in game and network conditions that can be accomplished in laboratory settings is necessarily restricted, and that humans are influenced by many other things than just following a strict rule of play, these diverging experimental findings are perhaps not that surprising. Still, they signal that a more detailed theoretical analysis of how behavior in coordination games is conditioned by the network in which actors are embedded might be informative also for the interpretation of the divergent empirical results. We will interpret some of these experimental findings in more detail in light of our theoretical implications in Sect. 6.

## Simulation of Coordination Games Played on a Network

In our simulation, actors interact in connected symmetric binary networks, where the number of actors varies between 2 and 25. In each round, actors play the same $2 \times 2$ coordination game with their neighbors, the actors with whom they are connected. Actors have a fixed strategy with respect to all their neighbors within a single round. After each round, actors can choose to update their choice of behavior in the coordination game in the next round. The simulation ends when convergence is reached (no actor wants to change behavior any more) or a given number of rounds (see below) have been played. After a simulation, we save the proportion of actors playing the behavior related to the payoff-dominant equilibrium, the payoffs in the constituent coordination game and the characteristics of the network. Across simulations, we vary the network (we ran simulations for 112,614 networks) and the payoffs (81 different payoff configurations). The details of the simulation are presented below.

### Sampled Networks and Network Characteristics

We only consider connected networks, with network sizes (the number of actors) varying from 2 to 25. For networks with size 2 through 8, we generated all 12,112 connected non-isomorphic graphs. For network sizes larger than 8, we generated all connected networks for smaller and larger numbers of ties and sampled networks from the set of networks with intermediate numbers of ties. For networks with sizes from 9 through 12, we generated roughly 10,000 networks per network size. For networks with sizes from 13 through 16, we generated roughly 5000 networks per network size, and for networks of size 17 through 25, we generated roughly 3800 networks per network size. Taken together, this gives us a set of 112,614 different networks. A more detailed description of the sampling of networks can be found in the Appendix.

We calculated several characteristics for each network:

- *Size*: the number of actors in the network.
- *Density*: the proportion of ties present in the network, which equals the number of ties divided by $(\hbox {size} \cdot (\hbox {size} - 1))/2$.
- *Degree*: the number of neighbors each actor has (an actor characteristic that we use to calculate further network characteristics).
- *Centralization*: the standard deviation in the degrees of the actors divided by the size of the network (cf. [29]; ‘heterogeneity’ in terms of Santos et al. [28]).
- *Segmentation*: comparing across all distances between pairs of actors in the network that are larger than 1,^1^ this is the proportion of distances that is 3 or larger. If all actors are directly connected, all distances are equal to 1 and the measure is defined as 0 [3].

Loosely speaking, we tried to capture ‘how many ties there are’ (density), ‘the extent to which there are actors with much more ties than others’ (centralization) and ‘the extent to which the network consists of strongly connected groups that are themselves loosely connected’ (segmentation). There are of course more possibilities to include network characteristics, but these are some of the most basic ones. Whereas these measures are of clear substantial interest, we also control for other network characteristics that affect the results, as will become clear in Sect. 3.

- *Maximal degree*: the largest degree that occurs in the network.
- *Proportion of actors with an odd number of neighbors*

### The Choice of Payoffs in the Constituent Game

The coordination game being played in each round of a simulation is the two-actor symmetric normal-form game as displayed in Table 1. Because $b < c < a < d$, (D, D) and (C, C) are both Nash equilibria. (C, C) is always the payoff-dominant equilibrium. If $a - b=d - c$, then the mixed equilibrium is risk-dominant, and if $a - b < d - c$, then (C, C) is both the risk-dominant and the payoff-dominant equilibrium. We focus on the more interesting case where $a - b > d - c$. In this case, (D, D) is the risk-dominant equilibrium and (C, C) the payoff-dominant equilibrium. That (D, D) is the risk-dominant equilibrium can easily be inferred by considering what actors would do as a best reply if the other actor would play D and C with equal probability. In our simulations, we chose $b = 0$ and $d = 20$ and varied $a$ and $c$ randomly between 0 and 20, while making sure that $a > c$ and $a + c >$ 20. Using integers for all payoffs, this gives 81 different payoff configurations.

**Table 1:** The constituent coordination game ($b < c < a < d$)

	D	C
D	$a, a$	$c, b$
C	$b, c$	$d, d$

For each network of size 2–12, we ran two simulations, each with a randomly chosen payoff configuration. For networks with sizes 13–25, we ran only one simulation with a randomly chosen payoff configuration. In sum, this leads to a total number of 165,428 simulations. Although this implies that we simulate either one or two payoff configurations per network structure, we chose to restrict our simulations in this sense because the number of networks is large enough to ensure that all payoff configurations are adequately represented in the simulation sample: All payoff configurations co-occur with all network characteristics.

### Behavior in One Round of Play and the Adaptation Strategy

In each round, an actor can choose between C and D, but we assume that actors must play their choice of behavior against *all* their neighbors: They cannot differentiate their behavior with respect to their neighbors. Note that this is similar to the way in which [14] and most others discussed in the introduction operationally define the freedom of choice of behavior that actors have. After each period, actors observe the percentage of their neighbors that played C and can decide to change their behavior for the following round.^2^ We ran separate simulations for two so-called adaptation strategies, i.e., two ways in which actors could adapt their behavior between rounds of play with their neighbors:

- [Adaptation Strategy 1: Myopic best reply] Each actor plays either C or D with probability 1 in round 1. In round $t + 1$, an actor plays what would have been the best reply against his neighbors’ play in round $t$.
- [Adaptation Strategy 2: Changing propensities] In round 1, each actor has a propensity to play C equal to 0.5. If his best reply against the behavior of his neighbors at time $t$ would have been D, he increases his propensity to play D with 0.1. If his best reply would have been C, he decreases his propensity to play D with 0.1. Clearly, propensities will be bounded between 0 and 1.^3^

We continued the simulation until convergence was reached or a specific number of rounds (depending on the adaptation strategy, see below) had been played.

For adaptation strategy 1 (in which behavior is completely deterministic), we calculated the average proportion of C choices after the process converged. Convergence was reached if, given the behavior of others, no actor wanted to change behavior anymore. For networks with at most 10 actors, we calculated this average proportion across all possible starting configurations of C and D behavior in a network $(2^{10} = 1024$ configurations). For network sizes $n$ larger than 10, we chose 1024 starting sets of playing C or D out of the $2^{n}$ possible starting configurations. The starting sets were chosen such that starting states with many Ds as well as with many Cs were guaranteed, while taking into account that the average probability to play C or D across the whole set of networks with a given size remained 0.5.^4^ Most of the time we found convergence to all-D, sometimes to all-C and sometimes to a heterogeneous equilibrium in which some play D and some play C (only in 1.6 % of the simulations all starting configurations within one network converged to the same state, namely all-D). In addition, because of the discrete and deterministic updating rule, sometimes a starting configuration led to flipping back and forth between two states (9.4 % of the times). When this occurred, we saved the average of Ds across these two states. It never occurred that there was a larger cycle of states in which the process got stuck.

In the case where actors’ behavior is not deterministic but based on propensities (adaptation strategy 2), we chose a starting position with propensities all equal to 0.5 (for all network sizes) and replicated the simulation 100 times. Convergence was reached when no actor wanted to change propensity any more, and at that point, we registered the percentage of actors who played C. This percentage we averaged across the 100 replications. Most of the time, these replications converged to an all-D or an all-C state. Only in 2.3 % of the replications, a heterogeneous equilibrium was reached. We set the maximal number of rounds within these replications to 1000, and for the smaller networks, convergence was always reached. We discovered later that convergence was not always reached within 1000 rounds for larger networks. We checked whether excluding the networks that did not always converge (this happened in less than 0.2 % of the networks, while it happened in less than 0.06 % of the networks more than four times out of 100 replications) changed our results, but this was not the case.

Taken together, this generates a data set with 165,428 ‘observations,’ where each observation consists of the average proportion of actors playing C across the replications at the end of a simulation, the payoff configuration that was used in the constituent game and the set of network characteristics as described above. This enables us to analyze how network characteristics (and choice of payoffs) relate to the likelihood that the payoff-dominant equilibrium is reached. Before we do that, we first present some analytic results.

## Analytic Preliminaries

Let ***A*** be the binary incidence or adjacency matrix representing the ties in the network of actors and ${{\mathbf {b}}}_{t}$ is the vector of behavior of actors at time $t$. $b_{t,i}$ = 1 means that actor $i$ played C with probability 1 at time $t$. Define $\hbox {RISK} := (a - b)/(a - b - c + d)$. $\hbox {RISK} > 0.5$ refers to the situation where (D, D) is the risk-dominant equilibrium. $\hbox {RISK} < 0.5$ refers to the situation where the risk-dominant and payoff-dominant equilibrium (C, C) coincide.

We now consider how the cooperation level depends on RISK. For the simulations on the basis of adaptation strategy 1, ‘myopic best reply,’ the following claims can be easily checked. Given a vector of behavior ${{\mathbf { b}}}_{t}$, actor $i$ changes to $b_{t+1,i} = 1 - b_{t,i }$ if and only if

$$\begin{aligned} (1-2b_{t,i} )\left( {\sum _{i\ne j} {A_{ij} \left( {(b-a)+b_{t,j} (a+d-b-c)} \right) } } \right) >0. \end{aligned}$$

Likewise, given a complementary behavioral vector $1 -{{\mathbf { b}}}_{t}$, actor $i$ changes to $b_{t+1,i}=b_{t,i }$ if and only if

$$\begin{aligned} (1-2b_{t,i} )\left( {\sum _{i\ne j} {A_{ij} \left( {(c-d)+b_{t,j} (a+d-b-c)} \right) } } \right) >0. \end{aligned}$$

Note that the only difference between the two equations is that the $(b - a)$ term that appears in the first equation changes into $(c - d)$ in the second equation. These inequalities have two important implications. Consider two complementary states ${{\mathbf { b}}}_{t}$ and $1 -{{\mathbf { b}}}_{t}$. In that case,

1. If $a - b = d - c$ (we then have $\hbox {RISK} = 0.5$), the two inequalities are exactly the same. This implies that given two simulations with complementary starting positions, across these two simulations, any two complementary states are equally likely to occur (the two simulations with complementary starting positions are each other’s mirror image). Consequently, in this situation, we expect an average percentage of actors playing C of 50 % at the end of the simulation runs: For each network converging to a percentage of actors playing C equal to $p$, there is a ‘mirror network’ that converges to a percentage of actors playing C of $1 - p$. In particular, this result does not depend on the network in which the simulation takes place, which implies that when $\hbox {RISK} = 0.5$, there are no effects of network characteristics whatsoever.
2. If $$\hbox {RISK} \ne 0.5$$, we see something similar. The two expressions above imply that when we compare two simulations in which the values of $$a - b $$ and $$d - c$$ are reversed (say, $$a - b = 1$$ and $$d - c = 5$$ in one simulation, and $$a - b = 5$$ and $$d - c = 1$$ in the other), the occurrence of a state in one of these simulations coincides with the occurrence of the complementary state in the other simulation. Thus, if the network is the same in both simulations, the number of times we find actors playing D in one simulation corresponds with the number of times they play C in the other simulation. As a consequence, to the extent to which the network facilitates playing C in one simulation, it facilitates playing D in the other simulation. Note also that when we interchange the values of $$a - b$$ and $$d - c$$, we change the value of RISK to $$1 - \hbox {RISK}$$. Taken together, this implies that if we would analyze network effects for $$\hbox {RISK} < 0.5$$, we should find exactly the reversed effects as the ones we will find for values of $$\hbox {RISK} > 0.5$$ (under the assumption, as we have here, that the population of networks that are considered is symmetric). Therefore, we can restrict our analyses below to values of $$\hbox {RISK} > 0.5$$ and can infer the network effects for $$\hbox {RISK} < 0.5$$.

In the simulations on the basis of adaptation strategy 2, ‘changing propensities,’ the arguments are basically the same. We can follow the line of argumentation as given for the myopic best reply adaptation strategy, because the same inequalities determine the probabilities to change propensities. Hence, also in this case, we can restrict ourselves to values of $\hbox {RISK} > 0.5$.

Next, we show that the relation between RISK and the cooperation level is a step function. Assume $a - b > d - c$, and hence that $\hbox {RISK} > 0.5$. An actor will increase his propensity to play C in a round of a simulation if and only if $\tfrac{\mathrm{Number \ of \ neighbors \ playing \ C{}^{}}}{\mathrm{Total \ number \ of \ neighbors}}>\tfrac{a-b}{a-b-c+d}>\tfrac{1{}^{}}{2_{}}$. This implies that if an actor has only one or two neighbors, the above equation is fulfilled if and only if *all* of his neighbors play C, irrespective of the precise values of the payoffs. Consequently, in this particular case, variations in RISK do not directly affect the propensity to play C of actors with one or two neighbors. Hence, should we happen to consider a network in which all actors have two neighbors or less, then the expectation to reach the (C, C) equilibrium does not depend on RISK, as long as $\hbox {RISK} > 0.5$. In a similar fashion, one can argue that for actors with three neighbors, it matters only whether RISK is larger or smaller than 2/3, and for actors with four neighbors, it matters only whether RISK is larger or smaller than 3/4. For actors with five neighbors, it matters whether RISK is smaller than 3/5, between 3/5 and 4/5, or larger than 4/5. Note that these RISK thresholds are of importance in the complete network and depend crucially on the maximal number of neighbors (the maximal degree) in the network.

As one example, consider the case where the maximal degree in a given network equals 6. The threshold values are 1/2 (for those with two neighbors), 2/3 (for those with 3 neighbors), 3/4 (for those with 4), 3/5 and 4/5 (for those with 5), and 4/6 and 5/6 (for those with 6 neighbors).

As Table 2 clarifies, for networks with a maximal degree of 6, the simulation results are stable in between the RISK thresholds. In general, based on the same logic, the simulation results are stepwise constant for all networks (and hence for all our simulation data). Note that when the number of neighbors one has is even, it is generally more difficult ‘to pass a threshold.’ This is because in our simulation, for any given number of neighbors, the first threshold of interest is the first proportion that can occur that is larger than 50 %. For even numbers, this tends to be a larger number. For instance, an actor needs 3 neighbors out of 4 playing C before the actor switches to C, but with a total of 3 neighbors, the actor would have needed only 2 out of 3, or with 5 neighbors only 3 out of 5. Therefore, if we are dealing with a network with a lot of actors who have an even number of neighbors, it will be more difficult to reach the (C, C) equilibrium.

**Table 2:** RISK thresholds in the simulations for networks with maximal degree at most 6

RISK threshold	Number of neighbors for which threshold is relevant
1/2	2, 4, 6
3/5	5
2/3	3, 6
3/4	4
4/5	5
5/6	6

Because the proportion of neighbors that behaves the same or differently as a focal actor can only take the values mentioned, the expected percentage of C choices does not vary between these RISK thresholds

## Simulation Results

We now estimate the relation between the proportions of times actors play C after convergence as dependent on the network characteristics and the payoffs. The analyses for the two different adaptation strategies, ‘myopic best reply’ versus ‘changing propensities,’ are in perfect concordance. We therefore only report the analyses for the simulation based on the propensities.

For reasons outlined in the previous section, we chose to analyze the relation between the network characteristics and the proportion of times actors play C separately for different values of RISK (as opposed to including the value of RISK as a covariate). The next question is for which values of RISK these steps are made in our simulations. We ran our analyses under the restrictions that $b = 0, d = 20, b < c < a < d$, and $a+c > 20$. Given these restrictions, there are $17 + 15 + 13 + 11 + 9 + 7 + 5 + 3 + 1 = 81$ ways to choose $a$ and $c$. In fact some of these choices lead to the same value of RISK. For instance, $(a, c) = (16, 12)$ and $(a, c) = (18, 11)$ both correspond to $\hbox {RISK} = 2/3$. It turns out that there are 76 different RISK values larger than 0.5 in our simulations. To keep matters tractable, we group some of the RISK categories that are created using these 76 thresholds together, based on a preliminary analysis predicting the proportion of C choices on the basis of dummies for all but one of the RISK values. This revealed where the most substantial steps in the described step function were made. On the basis of these preliminary results, we divided the RISK values in 14 categories (cf. Table 3).

**Table 3:** Average percentage of C choices and proportion convergence to heterogeneous state, per RISK category

RISK category	Interval per RISK category	Average percentage of C choices	Proportion convergence to heterogeneous state
1	0.500–0.526	26.9	3.8
2	0.526–0.530	26.1	4.1
3	0.530–0.534	25.7	4.1
4	0.534–0.540	24.8	4.0
5	0.540–0.546	23.5	3.9
6	0.546–0.552	22.2	4.1
7	0.552–0.556	20.5	3.6
8	0.556–0.567	18.7	3.9
9	0.567–0.572	15.5	3.7
10	0.572–0.599	12.9	3.6
11	0.599–0.601	8.2	2.8
12	0.601–0.666	3.7	2.5
13	0.666–0.667	0.9	0.26
14	0.667–1.000	0.2	0.09

We start to see network effects around $\hbox {RISK} = 10/19$ ( ${=}$ 0.526). Actors with 19 neighbors or more are the sole cause of this effect. Therefore, our first interval of RISK values is (0.5, 0.526). The next cutoff value is reached for actors with 17 neighbors at 9/17, leading to a second interval [0.526, 0.53). The other cutoff levels are likewise related to actors with a specific number of neighbors who become important as soon as that particular level of RISK is passed. Explaining the proportion of actors playing C with this limited number of categories hardly reduced the explained variance compared to the full model.^5^

Another reason why it makes sense to divide our simulation in several RISK categories is that *network effects turn out to depend to a considerable extent on the value of RISK*. In extreme cases (RISK close to 0.5 or closer to 1), network effects do not matter at all, but for specific values of RISK, the network effects get to be more important. By running our analyses per RISK category, we can study the variation in the sizes of the network effects while moving from low to high-risk situations.

The first results of our simulations can be seen in Table 3. Table 3 shows the proportion of actors that chooses C at the end of a simulation, averaged across RISK values within RISK categories and across the 100 replications per simulation. It appears that the proportion of times actors play C after convergence strongly decreases with RISK. Thus, the more risky the payoff-dominant equilibrium is, the less likely that the strategy related to that equilibrium is chosen. Even for RISK values relatively close to 0.5, the propensity that actors play C after convergence is considerably lower than the propensity that they play D. Note that in experimental research, a considerable likelihood, around 25 %, for playing C seems to be realized for larger values of RISK than in our simulation. Without network context, Friedman [16] finds around 20 % of C behavior for RISK = 2/3 (see also [32]). Berninghaus et al. [4] find even more C behavior for $\hbox {RISK} = 7/10$ in a network context. We return to this issue in our conclusion section. Only a limited percentage of the simulations converged to a state in which the behavior of the actors remains heterogeneous. It also seems that this percentage is relatively stable until $\hbox {RISK} = 0.6$ (category 11) where it drops down. A second drop occurs at $\hbox {RISK} = 2/3$ (category 13).

We further analyze the data produced by the simulations using standard linear regression methods.^6^ The summary statistics of our variables are presented in Table 4, starting with our two target variables: (1) the proportion of actors who play C after convergence (averaged across 100 replications) and (2) the proportion of the 100 replications that converges to an equilibrium in which behavior is heterogeneous (some actors play D while others play C). The independent variables are the network characteristics that we defined above: density, segmentation, centralization, size, proportion of actors with an odd number of neighbors and the maximal degree.^7^

**Table 4:** Summary statistics of key dependent and independent variables (165,428 observations)

Variable	Mean	St. Dev.	Minimum	Maximum
Proportion C	0.11	0.13	0	0.62
Proportion heterogeneous	0.02	0.08	0	0.88
Density	0.56	0.23	0.08	1
Centralization	0.30	0.10	0	0.87
Segmentation	0.14	0.21	0	0.89
Size	13.11	4.97	2	25
Percentage actors with odd neighbors	0.51	0.16	0	1
Maximal degree	9.04	4.49	1	24

As we have argued above, there are no effects of networks for $\hbox {RISK} = 0.5$, the results are symmetric around $\hbox {RISK} = 0.5$, and the cooperation level is dependent on RISK as a step function. We split the data in three intervals of RISK values: between 1/2 and 3/5, between 3/5 and 2/3 and larger than 2/3. There is still variation in effect sizes of network characteristics predicting the average propensity to play C within these intervals, but it nevertheless makes the varying importance of network effects clearer (see Table 5). We show some more details on variation within RISK categories later. Considering the propensity to end in an equilibrium with heterogeneous behavior, there is hardly any further variation in the effect sizes of network characteristics for different RISK categories than the differences between the three categories as distinguished in Tables 5 and 6.

**Table 5:** Linear regression analyses on the average proportion of actors playing the payoff-dominant equilibrium for different sizes of RISK and controlling with dummies for relevant RISK categories

	$1/2 < \hbox {RISK} < 3/5$	$3/5 \le \hbox {RISK} < 2/3$	$\hbox {RISK} \ge 2/3$
Density	0.20	0.020	0.007
Segmentation	$-$0.10	0.094	0.006
Centralization	0.086	($-$0.004)	0.009
Size/25	0.049	$-$0.14	$-$0.002
Proportion actors with odd neighbors	0.30	0.056	($-$0.001)
(Maximal degree)/24	$-$0.093	0.059	$-$0.002
Constant	(0.001)	0.081	0.004
$R^{2}$	0.56	0.27	0.14
$R^{2}$ (only dummies RISK categories)	0.21	0.044	0.099
Number of observations (networks)	75,777 (64,612)	34,473 (32,136)	55,178 (49,297)

Standard errors corrected for clustering within the same network, all coefficient are significant at $p < 0.001$ except for the coefficients between brackets

**Table 6:** Linear regression analyses on the proportion convergence to an equilibrium with heterogeneous behavior for different sizes of RISK and controlled with dummies for the relevant RISK categories

	$1/2 < \hbox {RISK} < 3/5$	$3/5 \le \hbox {RISK} < 2/3$	$\hbox {RISK} \ge 2/3$
Density	$-$0.037	($-$0.017)	$-$0.005
Segmentation	0.32	0.27	0.013
Centralization	$-$0.078	$-$0.11	($-$0.001)
Size/25	$-$0.11	$-$0.13	$-$0.012
Proportion actors odd neighbors	0.095	0.075	0.002
(Maximal degree)/24	0.11	0.12	0.015
Constant	(0.008)	(0.019)	0.003
$R^{2}$	0.56	0.46	0.062
$R^{2}$ (only dummies RISK categories)	0.0003	0.0002	0.0029
Number of observations (networks)	75,777 (64,612)	34,473 (32,136)	55,178 (49,297)

Standard errors corrected for clustering within the same network, all coefficients are significant at $p < 0.001$ except for the coefficients between brackets

**Fig. 1:**
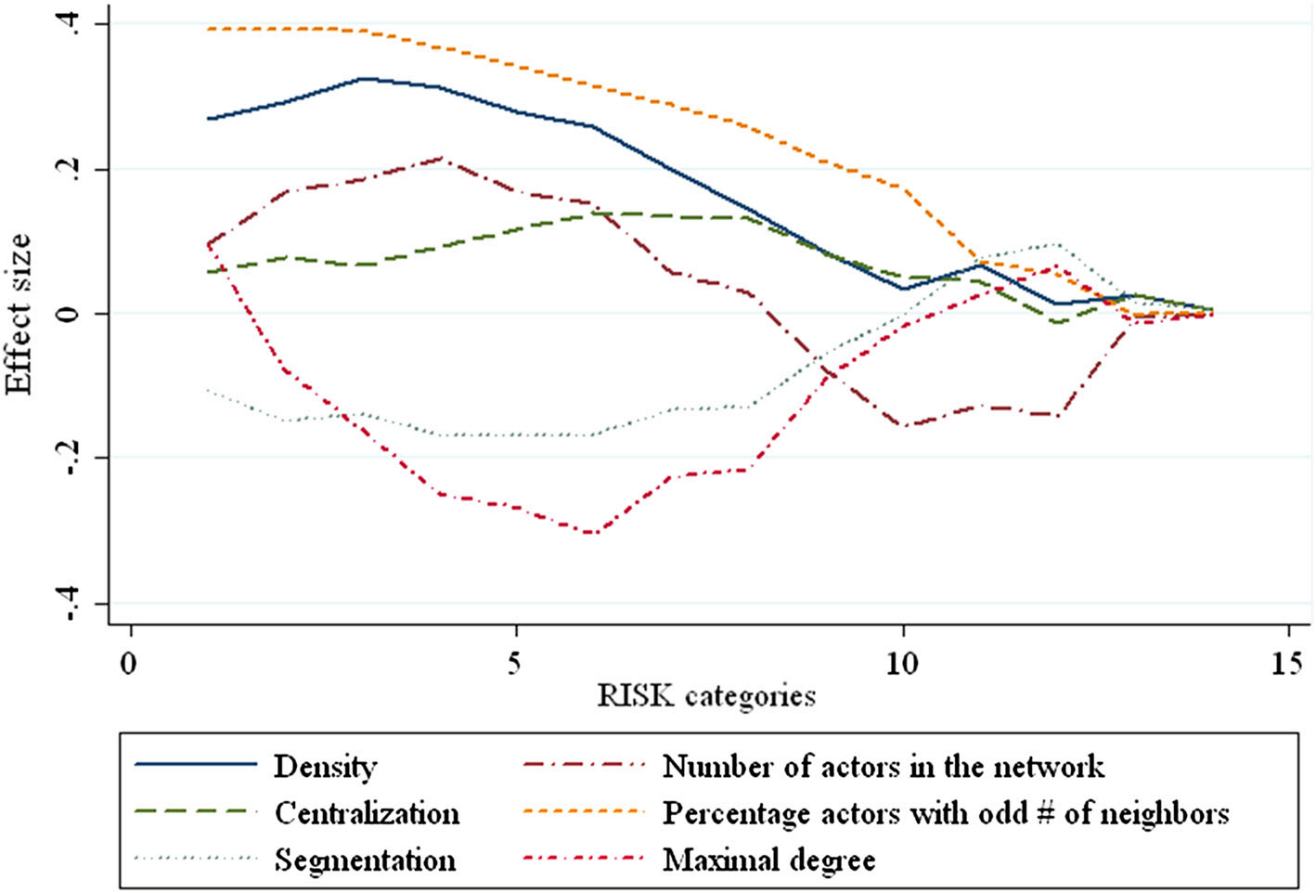
Effects of network characteristics on the average proportion of actors playing the payoff-dominant equilibrium for fourteen RISK categories. Effect size shows how much the likelihood of reaching the payoff-dominant equilibrium increases with a one-unit increase in the related independent variable for a specific RISK category. Note that the scale on the *x*-axis is not linear in RISK, because the RISK categories are not equidistant (cf. Table 3)

Table 5 shows that network density has a positive effect on playing C, but the size of the effect decreases with increasing RISK. Segmentation has a negative effect if RISK is between 1/2 and 3/5, but a positive effect if RISK is larger than 3/5. This can be explained as follows. The effect of segmentation has two sides: segmentation inhibits the diffusion of a behavior throughout the whole network, while it helps to maintain a given kind of behavior in a small part of the network while others choose the other behavior. Therefore, if C behavior starts to spread in the network, segmentation will reduce the probability that diffusion takes over the whole network. If D is the more dominant behavior, segmentation helps to maintain C in a small part of the network. Clearly, these two effects work in different directions, and the second effect becomes more important when the likelihood that D takes over the network becomes larger, that is, if RISK is larger. This argumentation is also confirmed by the fact that the positive effect of segmentation disappears if we control for the proportion of times the network converged to an equilibrium with heterogeneous behavior. The effect of centralization is positive but a bit smaller than the effect of density and it almost vanishes for $\hbox {RISK} > 3/5$. As expected, the effect of having an odd number of neighbors is positive because in that case, the probability to have, by chance, a majority on your side is larger. To prefer playing C, it is necessary to have a majority of neighbors playing C, while actors prefer to play D if neighbors are equally divided among playing D or C. We do not have adequate interpretations for the effects of network size and maximal degree. The effect of size is positive for RISK close to 0.5 and becomes negative for larger values of RISK. The effect of maximal degree is negative for RISK close to 0.5 and becomes positive for larger values of RISK. Notice that the explained variance decreases rapidly with RISK as well. If RISK becomes larger, the likelihood that the network can actually help to prevent behavior from moving to the risk-dominant equilibrium reduces quickly. Finally, we graph the regression coefficients of the six network characteristics in Fig. 1, where the analyses are done for each RISK category separately. This figure shows that in particular in the first 10 RISK categories, the effects change with RISK, but mostly stay consistently either positive or negative (network size is the exception). The figure also demonstrates again that the network effects decrease dramatically if RISK is larger than 2/3. All the network effects also seem to start decreasing for RISK approaching 0.5. Note that in all likelihood, this is not a spurious effect, since all network effects are 0 at $\hbox {RISK} = 0.5$.

In Table 6, we show three regression analyses predicting the probability to converge to a state in which behavior is heterogeneous.^8^ As was shown in Table 3, this probability is always low. Surprisingly, there is hardly any effect of density on expected heterogeneity of behavior, and it shows a pattern that is difficult to interpret. Given the problems to interpret significance in these simulated data and the relatively low $t$ values for the coefficient, we maintain the claim that there is hardly any evidence to support a substantial effect of density here. By far, the most important predictor is segmentation of the network, and it has, as expected, a positive effect on persistent heterogeneity of behavior. The proportion of actors with an odd number of neighbors also has a substantial positive effect. Centralization has a consistent negative effect on heterogeneity of behavior. Again, both the size of the network and the maximal degree of the network have small and difficult to interpret effects. Given that these hardly contribute to the explained variance, we will not try to interpret them in more detail. As in Table 5, we see that the explained variance decreases with increasing RISK. The last column in Table 6 once again shows that we need to be conservative in interpreting significance here, given that we have six highly significant effects although all the effects are very small and together explain only 6 % of the variance in the dependent variable.

## Sensitivity Analyses

Given the issues with interpreting significance as well as that we would like to know whether or not our results depend on the precise sample of networks we have drawn, we ran a set of additional analyses to check the robustness of our results, none of which lead to substantial differences in our results. By this, we mean that the statistical significances of effects of variables as depicted in Tables 5 and 6 are not affected by these alternative analyses and that the effect sizes are similar. We first ran some analyses where we weighted the observations in different ways. First, using variance-weighted least-squares regression, we checked whether it made a difference if we weight observations with the reverse of the variance that they had within the 100 replications. The rationale behind this is that if there is considerable variance across replications with respect to the proportion of actors that play C, one would expect that regression analysis does worse in predicting the average proportion for such observations (as compared to observations in which the variance is small). Second, we considered that every network in our data represents an isomorphism group of networks. One could argue that the larger this isomorphism group is, the more networks this particular network represents and, thus, the higher the weight that this particular network should have in the analyses. Therefore, we reran our regression analysis with the size of the isomorphism group that the networks represent as an observation weight. An issue with this weighting scheme is that the variation in network characteristics decreases and, as a consequence, effects become somewhat smaller. Third, we ran regression analyses for all RISK values separately (not only the 14 categories we described above), to make sure that effects do not depend on our choice of categories. Fourth, we split the data in subgroups that are more homogeneous in terms of network size to see whether the network effects strongly depend on the size of the network. Fifth, because the target variable equals 0 for a large proportion of the observations, especially when RISK is large, we ran regression analyses without the observations in which the proportion was equal to 0. As mentioned above, our results are consistent across all five alternative implementations of our analyses.

## Conclusion and Discussion

In the majority of networks, we find that actors eventually play the behavior that goes with the risk-dominant equilibrium in the $2\times 2$ coordination game. This was to be expected from the general result of Young [38] that in the long run, everyone is expected to play risk-dominant behavior. A new and important finding is that the structure of the networks affects the likelihood to converge only for values of RISK closely above and below 0.5 (when $0.33 < \hbox {RISK} < 0.67$ and $\hbox {RISK} \ne 0.5$). The most interesting case arises when $\hbox {RISK} > 0.5$, because only then networks can facilitate emergence to the payoff-dominant equilibrium. In this range, we find that density and centralization of a network have a positive effect on the propensity to reach the payoff-dominant equilibrium, while segmentation has a negative effect. In addition, our analyses on the state to which the network converges show that in a relatively small band of payoffs, the more segmented and less centralized networks (among the set of relatively small networks that we considered) are more likely to remain heterogeneous. Network density does not have an effect on the likelihood of behavior to converge to a homogenous state.

The important advantage compared to earlier work is that we identify a set of network characteristics that affect coordination behavior in networks while controlling for simultaneously varying other network characteristics. Santos et al.’s [28] finding that efficient coordination is more likely in heterogeneous networks is in line with our finding that centralization promotes efficient coordination, but we show that other types of heterogeneity in the network, such as segmentation, can have *negative* effects on reaching efficient coordination. Our results also provide alternative explanations for why previous theoretical papers in which sets of large networks are compared often do not find network effects (cf. [25–27, 30]). As we show for smaller networks, network effects are relevant only in a limited part of the payoff parameter space, but in this space, they do exist. In most of the papers comparing sets of (large) networks, a large range of payoffs is considered and when one compares results across this whole parameter space, network effects might indeed appear small or nonexistent. A second alternative explanation is that in the papers in this tradition, usually a few sets of networks are compared, for instance, scale-free networks and regular lattices, while the structural differences between these networks are multi-dimensional. Clearly, scale-free networks are more centralized than regular lattices, but they could also be more segmented (or differ in other network characteristics). Given that these two structural network characteristics have opposing effects according to our analyses, these two effects might cancel out by comparing a set of scale-free networks with a set of regular lattices. On the other hand, any differences found between, say, scale-free networks and regular lattices could have been due to multiple differences in characteristics of the network structure. We consider the identification of this issue as one of the main steps ahead of this contribution.

The positive effect of density on reaching the payoff-dominant equilibrium is consistent with the neighborhood size effect found in Berninghaus and Schwalbe [5] because in their models, a network is denser if neighborhood size increases. Our model gives a somewhat more elaborate underpinning of this effect in Berninghaus and Schwalbe [5], as in their case, their result was based on a restricted set of networks (only lattices were considered). There are also some notable differences with previous research in terms of predictions for empirical tests. Given that our model predicts that networks with odd neighborhood sizes are more likely to evolve to the payoff-dominant equilibrium than networks with even neighborhood sizes, we predict that the effect of neighborhood size is *not* monotonic. For instance, given the coefficients in our model, we predict that three-person neighborhoods converge to the payoff-dominant equilibrium more easily than four-person neighborhoods. Unfortunately, this result cannot be tested with the kinds of experiments as reported in Berninghaus et al. [4], because, probably coincidentally, only even-numbered neighborhoods were used in that case.

Comparing our results more closely with the experimental results by Berninghaus et al. [4], it is striking that although they use a rather high RISK value (0.7), they do find convergence to the payoff-dominant equilibrium as well as large differences between network conditions. This contrasts with our theoretical finding that the differences between networks mainly occur at RISK values between 0.5 and 0.6. One potential explanation for this is that our simulation is based on actors who start to make random choices, while subjects in the laboratory might use starting propensities with a higher weight on playing the payoff-dominant-related behavior. An alternative explanation is that introducing altruism in the model by placing at least some positive weight on the payoff for the other person in a utility function of this person would already decrease the RISK value of the game in terms of utility (as compared to in terms of only the payoffs). Therefore, it may be reasonable for comparing our results with these and future experimental results to assume that the subjective risk for the participants to play the payoff-dominant equilibrium is lower than if one would purely consider the payoffs. As we mentioned in the introduction, Berninghaus et al. [4] also find that the payoff-dominant equilibrium is reached more easily in the closed triad than in the circle with eight actors. The main variation here is the change in the density of the network and this result clearly corresponds with the positive effect we find for density on reaching the payoff-dominant equilibrium. When one compares a lattice in which everybody plays with four neighbors, with a circle where subjects also play with four neighbors (both networks with a total of 16 nodes), the only network characteristic that varies is segmentation. Density, centralization, etc., are all constant. Our positive effect of segmentation for intermediate risk values could explain the higher probability of subjects playing C on the circle. Of course, this cannot be considered as conclusive evidence for our theory given that the subjective risk is unclear and the effect of segmentation also changes depending on the value of RISK. Nevertheless, our theoretical results seem to have at least some consistency with these empirical results.

The fact that network effects are limited to the range of payoffs where there is a slightly stronger attraction of the risk-dominant equilibrium than of the payoff-dominant equilibrium (or the other way round) might also be the explanation why Antonioni et al. [2] do not find an effect of cliquish (segmented) networks on convergence to the payoff or risk-dominant equilibrium and Frey et al. [15] do not find the network effects that are expected from our analyses. Again one issue might be that the subjective risk is not the same as the risk we consider purely based on monetary payoffs. A strong argument in favor of this explanation is that the payoff-dominant equilibrium remains a strong attractor even with RISK is considerably larger than 0.5. Both papers blame deviations from theoretical predictions also on the assumption of myopic best reply behavior and suggest that subjects might use more sophisticated behavioral strategies. Although in general we would prefer to stick to the more simple behavioral theory, we concede that making the behavioral assumptions more complex is an equally promising strand for further research in trying to understand the dynamics of coordination on networks better.

Besides the comparison with existing empirical research, we provide several new hypotheses on effects of network characteristics on both the playing behavior related to the payoff-dominant equilibrium and the likelihood that multiple norms persist after convergence. Testing some of these hypotheses empirically remains a challenge for future research. A likely theoretical extension would be to consider how conclusions would change if we do not only allow actors to change their ties but also to change their partners. For an overview on the literature on these dynamic networks, one can consult Dutta and Jackson [13]. Specific models on coordination games played on networks are studied by Jackson and Watts [19], Goyal and Vega-Redondo [17], Berninghaus and Vogt [6], Buskens et al. [8] and Tsvetkova and Buskens [31].

## Appendix: Sampling of Networks

Below you find a more detailed account of the number and kind of networks sampled. The networks are available for research purposes. For network sizes from 2 through 8, we use all connected non-isomorphic networks $(N = {12{,}112})$. Networks are generated using specialized software called Nauty version 2.2 (see [22]).

For networks size 9 through 25, we use all the connected non-isomorphic networks for densities (number of ties) for which the number of different networks is limited. For other densities, we choose a random sample of the connected networks, where random means that each tie had the same probability of being in the network.

In detail:

- Size 9: all networks with 8–10 or 26–36 ties; sample of 400 for networks with 11–25 ties.
- Size 10: all networks with 9–10 or 36–45 ties; sample of 300 for networks with 11–35 ties.
- Size 11: all networks with 10–11 or 46–55 ties; sample of 200 for networks with 12–45 ties.
- Size 12: all networks with 11 or 57–66 ties; sample of 200 for networks with 12–56 ties.
- Size 13: all networks with 69–78 ties; sample of 50 for networks with 12–68 ties.
- Size 14: all networks with 82–91 ties; sample of 50 for networks with 13–81 ties.
- Size 15: all networks with 96–105 ties; sample of 50 for networks with 14–95 ties.
- Size 16: all networks with 111–120 ties; sample of 50 for networks with 15–110 ties.
- Size 17–25:
  - $\bullet$ all networks with $\hbox {size} \cdot (\hbox {size} - 1)/2$ to $\hbox {size} \cdot (\hbox {size} - 1)/2 - 5$ ties.
  - $\bullet$ sample of 20 for networks with size $-1$ to $\hbox {size} \cdot (\hbox {size} - 1) - 6$ ties.

Total number of networks: 112,614.

## Footnotes

1 Distance is the minimal number of ties through which two actors can reach each actor, also called minimal path (see [35]: 110–111).

2 It would not make a difference to assume that actors observe the behavior of all their neighbors. Because actors cannot differentiate their behavior among their neighbors, their replies are necessarily based on the proportion of neighbors playing D or C anyway.

3 One could also use a slightly more sophisticated (and standard) adaptation strategy in which the propensity $p$ to play ‘X’ in round $t+ 1$ is reinforced through $p(t + 1) = p(t)+q (1 - p(t))$, with $q$ a learning parameter. The results are very similar.

4 To see how this was accomplished, suppose $n = 25$. We need to create 1024 binary strings of length 25, representing behavior of the 25 actors. Then for every $k$, with $k = 1, \ldots , 1024$, we first randomly select a binary string of length 15 and then complete it with the number $k$ in 10-digit binary representation.

5 Note that the cutoff value for actors with 18 neighbors is only at $10/18 = 0.556$, which is even beyond the cutoff levels for 15, 13 and 11 actors and is the same as for actors with 9 neighbors. This shows once again that when we compare actors with a given odd number of neighbors with actors with an even number of neighbors, actors with an odd number of neighbors are more likely to pass a threshold value of neighbors such that playing C is advantageous.

6 As our target variable is a proportion, one could consider a logit transformation to ensure that predicted proportions remain within the (0, 1) interval. As the results turned out to be substantially similar and the disadvantage is that this makes interpretation and comparison of the coefficients more complicated, we chose to present standard linear regression analyses.

7 We divided size by 25 and maximal degree by 24 in the analyses to make the effect sizes more comparable with the other variables that also vary between 0 and 1.

8 Again the results do not change substantially when we first logit-transform the target variable.
